# Advancing life course immunization in Ethiopia: A policy imperative for universal health coverage and equity

**DOI:** 10.1016/j.ghrp.2026.05.004

**Published:** 2026-05-29

**Authors:** Geteneh Moges Assefa, Michael Tarekegn, Kasahun Negash, Azmeraw Ayehu Tesfahun, Wendemagegn Yeshanehe, Muluken Desalegn Muluneh

**Affiliations:** a Amref Health Africa in Ethiopia, Ethiopia; b Development Management, Debre Berhan University, Ethiopia; c Selam Global Health Consultancy, Ethiopia

**Keywords:** Life course immunization, Universal health coverage, Equity, Ethiopia

## Abstract

Vaccination is a cornerstone of public health and a critical enabler of universal health coverage, yet Ethiopia’s immunization system remains largely oriented toward early childhood. Although childhood coverage for key antigens has improved substantially, recent evidence shows persistent zero-dose and under-immunization among children, uneven uptake of vaccines for adolescents, pregnant women, adults, and older adults, and missed opportunities even among populations living near health facilities. Life course immunization (LCI), defined by Immunization Agenda 2030 as vaccination across all ages and life stages through integrated health systems, offers a policy pathway to close these gaps. This policy brief argues that Ethiopia should institutionalize LCI as a routine primary health care function rather than relying on fragmented campaigns or childhood-focused platforms alone. The evidence points to interacting demand-side, supply-side, and health-system barriers, including limited awareness of vaccines beyond childhood, low perceived risk, concerns about side effects, gender-related decision-making constraints, weak adolescent and adult service platforms, and insufficient age-disaggregated monitoring. By embedding LCI into national immunization policy, Ethiopia can create repeated vaccination contact points, strengthen risk communication, improve accountability across age and risk groups, and reposition vaccination as a lifelong public good for equity and universal health coverage.

## Background

Vaccination currently prevents an estimated 3.5–5 million deaths each year and is a core primary health care and universal health coverage intervention.^1^ Ethiopia’s Expanded Programme on Immunization (EPI) has expanded steadily since the early 2000s, contributing to major progress in infant and childhood vaccination and reductions in vaccine-preventable diseases. However, routine immunization policy and delivery remain concentrated on early childhood, while platforms for adolescents, adults, older people, and high-risk groups are less systematically defined, financed, monitored, and linked to routine services.^2^, ^3^ This gap matters because vaccination beyond childhood protects pregnant women, adolescents, adults with occupational or chronic-disease risks, and older adults whose immunity, exposure patterns, or risk of severe disease differ from those of children.

The COVID−19 pandemic exposed the consequences of Ethiopia’s limited adult and adolescent vaccination infrastructure. Emergency campaigns enabled rapid scale-up of vaccination for adults and populations aged 12 years and above, but coverage remained uneven and implementation depended heavily on time-limited campaign delivery, partner-supported logistics, communication, and monitoring arrangements.^4^ Such ad hoc scale-up is not a sustainable substitute for a routine, financed, and accountable vaccination platform across age groups. The lesson is not only that COVID−19 created an opportunity for reform, but that Ethiopia needs routine adult and adolescent vaccination systems that can function beyond emergency campaigns.

Life course immunization (LCI), as articulated by Immunization Agenda 2030, reframes vaccination as a repeated preventive service across infancy, adolescence, pregnancy, adulthood, older age, and high-risk conditions, integrated with primary health care and other essential services.^2^ It differs from a traditional child-centred EPI model by extending target groups, delivery points, records, counselling, and accountability beyond infancy. It also differs from vertical campaigns because it requires routine financing, age-disaggregated information systems, trained providers, risk-based communication, and integrated delivery through platforms such as antenatal care, school health, chronic disease services, outreach, and community health systems.^2^, ^3^, ^5^ Thus, this policy brief aims to clarify the policy case for institutionalizing life course immunization in Ethiopia by synthesizing recent coverage evidence, identifying demand-side, supply-side, and health-system constraints, and proposing feasible actions for phased integration into primary health care and universal health coverage reforms.

## Evidence across the life course

Evidence from the 2025 Amref Health Africa–Federal Ministry of Health household survey provides recent policy signals on immunization gaps across age groups in Ethiopia.^6^ Among children aged 12–59 months, 9.1% were zero-dose and 11.2% were under-immunized; uptake in later life stages was also uneven, including HPV vaccination among eligible adolescent girls, tetanus–diphtheria vaccination among pregnant women, and COVID−19 vaccination among individuals aged 12 years and above.^6^ These patterns show that Ethiopia’s challenge is not limited to incomplete childhood vaccination. Rather, it reflects the absence of a routinely managed vaccination pathway across adolescence, pregnancy, adulthood, older age, and high-risk conditions.

The drivers of these gaps are partly demand-side. Caregivers and eligible vaccine recipients reported limited awareness of vaccination schedules, concerns about side effects, competing household priorities, and low perceived need for vaccination beyond childhood.^6^, ^7^ Gendered decision-making further constrains timely care-seeking, as women may have limited autonomy to seek vaccination for themselves or their children.^7^ These barriers suggest that LCI policy must do more than add new target groups or antigens. It must normalize vaccination as a lifelong preventive service through age-appropriate counselling, community engagement, and trusted risk communication.^8^

Supply-side constraints are equally important. More than half of zero-dose children lived within five kilometres of a health facility, indicating that physical proximity does not guarantee uptake when services are inconsistently offered, poorly communicated, or not linked to follow-up and reminder systems.^6^ For adolescents and adults, the gap is more structural: Ethiopia has fewer routine vaccination contact points outside antenatal care, school-based HPV delivery, and emergency COVID−19 vaccination. Weak documentation, limited provider preparation for adolescent and adult vaccination counselling, and missed opportunities during primary health care visits reduce the ability of facilities to convert service contact into vaccination.

At health-system level, coverage gaps reflect fragmented governance and limited accountability beyond childhood. Pastoralist and conflict-affected settings, particularly Afar, recorded zero-dose prevalence above 20%, showing that a uniform facility-based model is insufficient for mobile, insecure, or underserved communities.^6^, ^9^ At the same time, HPV and COVID−19 experience shows that campaign approaches can achieve rapid reach but often do not create durable financing lines, age-disaggregated records, routine delivery platforms, or accountability mechanisms for adolescent and adult vaccination. The available evidence is strongest on coverage and reported barriers, and weaker on timeliness, dropout, missed opportunities, and continuity across life stages. This evidence limitation itself supports the policy case for an LCI framework with routine age- and risk-disaggregated monitoring. Detailed survey methods, additional data-oriented evidence, and research-style methodological limitations are provided in Supplementary File 1.

## Policy recommendations

To operationalize life course immunization in Ethiopia while safeguarding gains in childhood vaccination, the following actions are recommended:

1. Embed LCI in national policy and governance: The Federal Ministry of Health should revise national immunization policy and strategic planning documents to explicitly define target age and risk groups across the life course, assign institutional responsibilities, and align LCI with primary health care, and UHC reforms. This would create a common accountability framework rather than leaving adolescent and adult vaccination to fragmented initiatives.
2. Integrate vaccination into existing service platforms: LCI should be delivered through established contact points, including the Health Extension Program, primary health care units, antenatal care, school health services, chronic disease clinics, outreach services, and emergency preparedness systems. These platforms matter because they create repeated opportunities to reach people beyond early childhood without building parallel delivery systems.
3. Strengthen workforce readiness through phased training: Health workers and Health Extension Workers should receive practical training in adolescent and adult vaccination, age-specific counselling, contraindication screening, adverse-event communication, missed-opportunity identification, and risk communication. Initial training should prioritize high-contact platforms such as antenatal care, school health, chronic disease follow-up, and community outreach.
4. Secure sustainable and sequenced financing: Ethiopia should develop domestic financing mechanisms for LCI, complemented by pooled procurement and coordinated partner support. Vaccine introduction should be sequenced using burden-of-disease, equity, cost-effectiveness, fiscal-space, and delivery-feasibility criteria, rather than introducing a broad adult-vaccine package all at once.
5. Modernize data and accountability systems: Immunization information systems should disaggregate coverage by age, sex, geography, pregnancy status, and risk group. Routine monitoring should also capture missed opportunities, dropout, and continuity of vaccination across life stages to support microplanning and performance review.
6. Build public demand and trust: Communication strategies should position vaccination as a lifelong preventive service, not only a childhood intervention. Community engagement, women’s groups, schools, professional associations, religious and community leaders, and trusted health workers should be used to address misinformation, fear of side effects, low perceived need, and gender-related decision-making barriers.

## Policy applicability and implementation considerations

Operationalizing life course immunization in Ethiopia will require phased implementation because primary health care facilities already face competing service-delivery, workforce, cold-chain, financing, and reporting demands. National policy can define priority age and risk groups, financing responsibilities, delivery platforms, and accountability indicators, but implementation should be adapted to regional context. In agrarian and urban settings, early integration may be feasible through antenatal care, school health services, chronic disease clinics, and primary health care units. In pastoralist, mobile, and conflict-affected settings, flexible outreach, community-based delivery, and microplanning will be critical. The main implementation risk is that LCI could become another vertical initiative unless it is embedded in routine PHC budgets, health worker training, age-disaggregated records, supervision, and performance review. These considerations support a gradual approach that begins with existing contact points and priority groups before expanding to a broader life course platform.

## CRediT authorship contribution statement

**Wendemagegn Yeshanehe:** Writing – review & editing, Validation. **Azmeraw Ayehu Tesfahun:** Writing – review & editing, Validation, Formal analysis, Conceptualization. **Kasahun Negash:** Writing – review & editing, Validation. **Michael Tarekegn:** Writing – original draft, Validation. **Geteneh Moges Assefa:** Writing – review & editing, Writing – original draft, Visualization, Validation, Methodology, Formal analysis, Conceptualization. **Muluken Desalegn Muluneh:** Writing – review & editing, Validation.

## Consent for publication

All authors have read and approved the final version of the manuscript and consent to its publication in *Global Health Research and Policy*.

## Ethics approval and consent to participate

Not applicable for this policy brief. This manuscript synthesizes evidence from published studies, policy documents, and supplementary evidence sources. The household survey used as one of the main source documents received approval from the Institutional Review Ethics Committee of the Federal Ministry of Health, Ethiopia, protocol code ETCO/Admin/550/25, approval date April 1, 2025, as reported in Supplementary File 1.

## Funding

The research was supported by the Mastercard Foundation under the Saving Lives and Livelihoods (SLL) initiative, grant number R252, implemented through Amref Health Africa in collaboration with the Africa Centres for Disease Control and Prevention (Africa CDC).

The funders had no role in the study design, data collection, analysis, interpretation of results, writing of the manuscript, or the decision to submit it for publication.

## Declaration of Generative AI and AI-assisted technologies in the writing process

During the revision of this manuscript, the authors used AI-assisted language support to improve clarity, structure, grammar, and formatting. The authors reviewed and edited all AI-assisted outputs, verified the accuracy of the content, and take full responsibility for the final manuscript.

## Declaration of Competing Interest

The authors declare that they have no known competing financial interests or personal relationships that could have appeared to influence the work reported in this article.

## References

[1] World Health Organization (2026 May 4). https://www.who.int/health-topics/vaccines-and-immunization/.

[2] World Health Organization (2020). https://www.who.int/publications/m/item/immunization-agenda-2030-a-global-strategy-to-leave-no-one-behind.

[3] Federal Ministry of Health Ethiopia (2021). https://repository.iphce.org/bitstream/handle/123456789/2941/cMYP%20%282021-2025%29.pdf?isAllowed=y&sequence=1.

[4] United Nations Ethiopia (2021 Nov 25). https://ethiopia.un.org/en/168766-ethiopia-launches-covid-19-vaccination-campaign-targeting-12-years-and-above-population.

[5] World Health Organization (2023).

[6] Assefa G.M., Damtew M.T., Negash K. (2026). Disparities and barriers to life-course vaccination in Ethiopia: evidence from a household survey. Hum Vaccin Immunother.

[7] Assefa G.M., Tarekegn M., Negash K. (2025). Gender dynamics in vaccine acceptance and hesitancy among primary caregivers in Ethiopia: a mixed-methods study. Vaccines.

[8] Aborode A.T., Fajemisin E.A., Ekwebelem O.C. (2021). Vaccine hesitancy in Africa: causes and strategies to the rescue. Ther Adv Vaccin Immunother.

[9] Scobie H.M., Blau D.M., Vora N.M. (2023). Zerodose children: global landscape and path forward. Vaccine.

